# A banana aquaporin gene, *MaPIP1;1*, is involved in tolerance to drought and salt stresses

**DOI:** 10.1186/1471-2229-14-59

**Published:** 2014-03-08

**Authors:** Yi Xu, Wei Hu, Juhua Liu, Jianbin Zhang, Caihong Jia, Hongxia Miao, Biyu Xu, Zhiqiang Jin

**Affiliations:** 1 Hainan Key Laboratory of Banana Genetic Improvement, Haikou Experimental Station, Institute of Banana, Chinese Academy of Tropical Agricultural Sciences, Yilong W Road. 2, Longhua County, Haikou City, Hainan Province 570102, People’s Republic of China; 2 Key Laboratory of Biology and Genetic Resources of Tropical Crops, Institute of Tropical Bioscience and Biotechnology, Chinese Academy of Tropical Agricultural Sciences, Xueyuan Rd. 4, Longhua County, Haikou City, Hainan Province 571101, People’s Republic of China

**Keywords:** Aquaporin, Banana, Drought stress, Salt stress

## Abstract

**Background:**

Aquaporin (AQP) proteins function in transporting water and other small molecules through the biological membranes, which is crucial for plants to survive in drought or salt stress conditions. However, the precise role of *AQPs* in drought and salt stresses is not completely understood in plants.

**Results:**

In this study, we have identified a *PIP1* subfamily *AQP* (*MaPIP1;1*) gene from banana and characterized it by overexpression in transgenic *Arabidopsis* plants. Transient expression of MaPIP1;1-GFP fusion protein indicated its localization at plasma membrane. The expression of *MaPIP1;1* was induced by NaCl and water deficient treatment. Overexpression of *MaPIP1;1* in *Arabidopsis* resulted in an increased primary root elongation, root hair numbers and survival rates compared to WT under salt or drought conditions. Physiological indices demonstrated that the increased salt tolerance conferred by *MaPIP1;1* is related to reduced membrane injury and high cytosolic K^+^/Na^+^ ratio. Additionally, the improved drought tolerance conferred by *MaPIP1;1* is associated with decreased membrane injury and improved osmotic adjustment. Finally, reduced expression of ABA-responsive genes in *MaPIP1;1*-overexpressing plants reflects their improved physiological status.

**Conclusions:**

Our results demonstrated that heterologous expression of banana *MaPIP1;1* in *Arabidopsis* confers salt and drought stress tolerances by reducing membrane injury, improving ion distribution and maintaining osmotic balance.

## Background

Plant growth depends greatly on water absorption from the soil and the movement of water from the roots to other plant parts [1]. However, environmental stresses such as drought, salt and cold can lead to water loss in plants. Such environmental stresses severely affect plant growth and productivity worldwide. Translocation of water is an important process to maintain the ability to tolerate desiccation and high salt stresses [2-4]. In plants, water movement is controlled by both apoplastic and symplastic pathways [1]. When plants are experiencing abiotic stress, the symplastic pathway is efficient for transporting water across membranes [5-7], and the symplastic pathway is regulated mainly by members of the aquaporin family of proteins [8].

Aquaporins (AQPs) transport water as well as other small molecules such as glycerol, CO_2_ and boron through membranes [9-11]. Biological activities associated with AQPs are diverse and include seed germination, stomatal movement, cell elongation, reproductive growth, phloem loading and unloading and stress responses in plants [12,13]. Many genes encoding AQP proteins have been identified from different plant species, including 35 from *Arabidopsis*[14], 33 from rice [15] and 36 from maize [16]. These orthologs can be subdivided into four groups characterized by highly conserved amino acid sequences and stereotypical intron positions within each group: the tonoplast intrinsic proteins (TIPs), the plasma membrane intrinsic proteins (PIPs), the nodulin-like plasma membrane intrinsic proteins (NIPs) and the small intrinsic proteins (SIPs) [17].

The expression and biological activities of AQPs are affected by a number of signals, including abiotic stresses, plant hormones and light [10,14,18-21]. The regulation and biochemical functions of *AQPs* in response to abiotic stresses are complex and not well understood. In a number of transgenic approaches, some *AQPs* have been demonstrated to confer tolerance to abiotic stresses [6,11,13,22-26]. For example, overexpression of *TaAQP8* results in increased root elongation under salt stress [25]. Tobacco *NtAQP1* is involved in improving water use efficiency, hydraulic conductivity, and yield production under salt stress [11]. However, overexpression of a distinct aquaporin, *HvPIP2;1*, leads to an increased transpiration rate and slightly decreased intrinsic water-use efficiency [27]. These attempts to use *AQPs* to improve crop tolerance to abiotic stresses have yielded contradictory results depending on the isoforms of *AQPs*. Therefore isoforms that are shown to confer improved physiological status under stress are of major importance in crop science.

Banana (*Musa acuminata* L.) is a large annual monocotyledonous herbaceous plant found in tropical and subtropical climates, and is one of the most popular fresh fruits enjoyed worldwide. Because banana has shallow roots and a permanent green canopy, it is especially sensitive to conditions that lead to water deficit [28,29]. A better understanding of the mechanisms employed by banana plants to tolerate abiotic stresses will be helpful for increasing crop production and quality of this economically valuable fruit. In banana, only one aquaporin gene, *MusaPIP1;2*, has been characterized as a positive factor in abiotic stress tolerance. Transgenic plants overexpressing *MusaPIP1;2* constitutively exhibited better abiotic stress survival characteristics including lower malondialdehyde content, elevated relative water content, elevated proline levels and a higher photosynthetic efficiency relative to controls under different abiotic stress conditions [29]. In our previous study, a transcript displaying upregulated expression at the early stage of post-harvest banana ripening was identified by cDNA microarray [30]. Sequence analysis suggested that this cDNA fragment exhibited high similarity to *AQP* genes from other plant species. In this study, a full-length cDNA encoding MaPIP1;1 was cloned and characterized. We investigated the function of *MaPIP1;1* during drought and salt stresses, which will lead to increased understanding of the mechanisms of environmental stress tolerance employed by plants.

## Results

### Banana *MaPIP1;1* encodes a PIP1-subfamily aquaporin

A cDNA fragment was identified by cDNA microarray from genes that were differentially expressed at the early stage of post-harvest banana ripening and the full-length cDNA, designated as *MaPIP1;1* (GenBank: KC969669), was obtained using the rapid amplification of cDNA ends (RACE) method. The full-length *MaPIP1;1* cDNA is 1123 bp in length with a 861 bp open reading frame (ORF) that encodes 286 amino acids. BLASTX analysis demonstrated that *MaPIP1;1* had 94% sequence identity with *HcPIP1* from *Hedychium coronarium* and *OsPIP1;2* from *Oryza sativa Japonica Group*. The predicted *MaPIP1;1* protein has a highly conserved amino acid sequence (‘HINPAVTFG’) characteristic of the MIP family, six putative transmembrane helices and two ‘NPA’ motifs (Additional file 1: Figure S1). Phylogenetic analysis of MaPIP1;1 with other AQPs from *Arabidopsis* and rice that MaPIP1;1 is close to PIP1 subfamily (Additional file 1: Figure S2). These results suggest that the *MaPIP1;1* gene cloned in this study is a member of the *PIP1* subfamily in banana.

### MaPIP1;1 localizes to the plasma membrane

**Figure 1:**
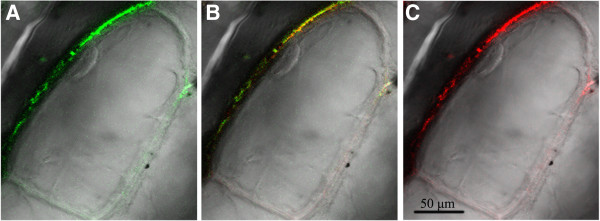
**Subcellular localization of MaPIP1;1 fused with GFP.** MaPIP1;1::GFP and plasma membrane-localized maker pm-rk were transiently co-expressed in onion epidermal cells and visualized with fluorescence microscopy after 48 h. **(A)** Fluorescence image of an epidermal cell expressing the p35S-MaPIP1;1::GFP fusion protein. **(B)** Merged fluorescence image of an epidermal cell expressing the p35S-MaPIP1;1::GFP fusion protein and pm-rk marker. **(C)** Fluorescence image of an epidermal cell expressing the pm-rk.

To determine the subcellular localization of the MaPIP1;1 protein, its ORF was introduced into pCAMBIA1304-GFP vector upstream of the *GFP* gene to create a MaPIP1;1-GFP translational fusion construct. The MaPIP1;1-GFP fusion and the plasma membrane-localized maker pm-rk were co-expressed in onion epidermal cells by particle bombardment and we observed that the green fluorescence MaPIP1;1-GFP and red pm-rk were both confined to the plasma membrane (Figure 1). These results indicate that MaPIP1;1 is targeted to the plasma membrane.

### Expression of *MaPIP1;1* in different banana organs and after various stress treatments

**Figure 2:**
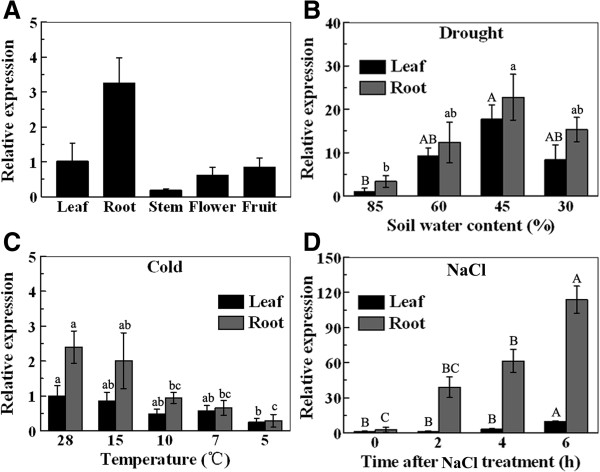
***MaPIP1;1 *expression in different banana organs (A) and in leaves and roots with stress treatments (B,C,D).** Data are means ± SE of n = 3 biological replicates. Means denoted by the same letter do not significantly differ at *P* < 0.05 as determined by Duncan’s multiple range test.

To investigate the expression of *MaPIP1;1* in different banana organs, total RNA was extracted from leaves, roots, stems, flowers and fruits, converted to cDNA and subjected to real-time quantitative polymerase chain reaction (qRT-PCR) analysis. *MaPIP1;1* transcripts were detected in all organs examined and the gene was most abundantly expressed in roots (Figure 2A). To determine the transcriptional response of *MaPIP1;1* to abiotic stress, various stress treatments were applied to banana plants. The results indicated that the expression of *MaPIP1;1* was induced in leaves and roots after salinity stress and simulated drought treatments. The highest expression levels of *MaPIP1;1* were observed when banana seedlings were treated with NaCl for 6 h and at a soil water capacity at 45% (Figure 2B and 2D). However, the expression of *MaPIP1;1* in leaves and roots was inhibited by chilling treatment (Figure 2C). Taken together, these results suggest that *MaPIP1;1* transcript levels were affected by various stress treatments.

### Phenotypic analysis of *MaPIP1;1* overexpressing *Arabidopsis* transgenic lines

**Figure 3:**
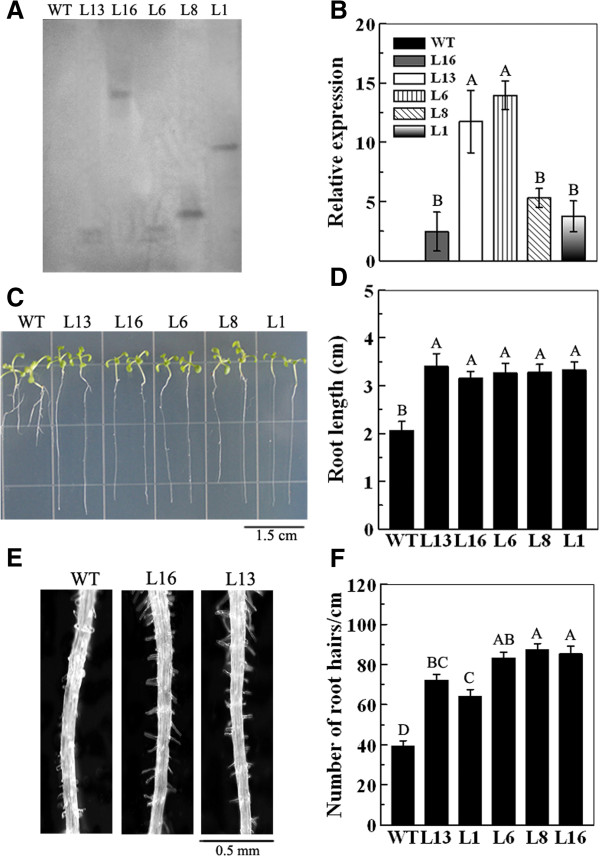
**Characterization of *MaPIP1;1*-overexpressing lines in *Arabidopsis*.** Leaves from four week-old plants were sampled to detect the *MaPIP1;1* copy number **(A)** and the expression of the transgene **(B)**. Photographs **(C)** and statistical analyses **(D)** of primary root length of WT and transgenic lines under normal conditions. Photographs **(E)** and statistical analyses **(F)** of root hairs of WT and transgenic lines under normal conditions. Data are means ± SE of n = 4 biological replicates. Means denoted by the same letter do not significantly differ at *P* < 0.05 as determined by Duncan’s multiple range test.

To further understand the role of *MaPIP1;1 in planta*, *MaPIP1;1* was introduced into pCAMBIA1304 vector under the control of the 35S promoter. After floral-dip transformation of *Arabidopsis*, five hygromycin-resistant transgenic lines from the T_3_ generation were investigated by Southern analysis. These results showed that *35S::MaPIP1;1–13* (L13) and *35S::MaPIP1;1–6* (L6) lines each integrated two copies of the *MaPIP1;1* transgene, while *35S::MaPIP1;1–16* (L16), *35S::MaPIP1;1–8* (L8) and *35S::MaPIP1;1–1* (L1) lines each integrated one copy of *MaPIP1;1* (Figure 3A). The expression levels of *MaPIP1;1* in the transgenic lines were also monitored. L13 and L6 exhibited higher levels of *MaPIP1;1* expression than the other transgenic lines, which is consistent with the copy number of *MaPIP1;1* determined by Southern analysis (Figure 3B). Transgenic *MaPIP1;1* overexpression lines exhibited longer primary root length, fewer emerged lateral roots and more abundant root hairs than untransformed controls (Figure 3C- 3 F; Additional file 1: Figure S3). These results suggest that *MaPIP1;1* overexpression influences root development under typical *Arabidopsis* growth conditions.

### Overexpression of *MaPIP1;1* enhances tolerance to salt stress

**Figure 4:**
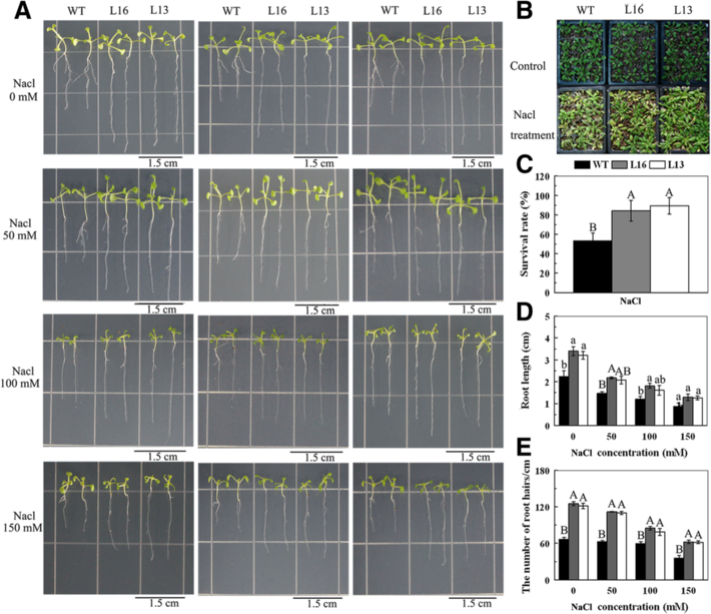
**Roponse to salt stress of *MaPIP1;1*-overexpressing *Arabidopsis *plants.** Photographs **(A)** and statistical analyses **(D)** of the primary root length of WT and transgenic lines under normal or saline conditions. Photographs **(B)** and survival rates **(C)** of WT and transgenic mature plants grown under saline conditions. **(E)** The number of root hairs of WT and transgenic lines under normal or saline conditions. Data are means ± SE of n = 4 biological replicates. Means denoted by the same letter do not significantly differ at *P* < 0.05 as determined by Duncan’s multiple range test.

To investigate the role of *MaPIP1;1* during salt stress, wild-type (WT) *Arabidopsis* and *MaPIP1;1* overexpression lines were subjected to salinity treatments. Root growth was enhanced in the transgenic lines compared to WT seedlings under control and high salt conditions. In NaCl conditions ranging from 50 mM to 150 mM, the transgenic seedlings exhibited reduced suppression of primary root length and more abundant root hairs than did WT seedlings (Figure 4A, 4D and 4E). Furthermore, when mature *Arabidopsis* plants were subjected to 350 mM NaCl treatment for 15 d in soil, the transgenic lines exhibited better growth and a higher survival rate than WT plants (Figure 4B and 4C). These results indicate that *MaPIP1;1* overexpressing transgenic lines were more tolerant to salt stress than WT *Arabidopsis*.

### Overexpression of *MaPIP1;1* reduces MDA content and IL under salt stress

**Figure 5:**
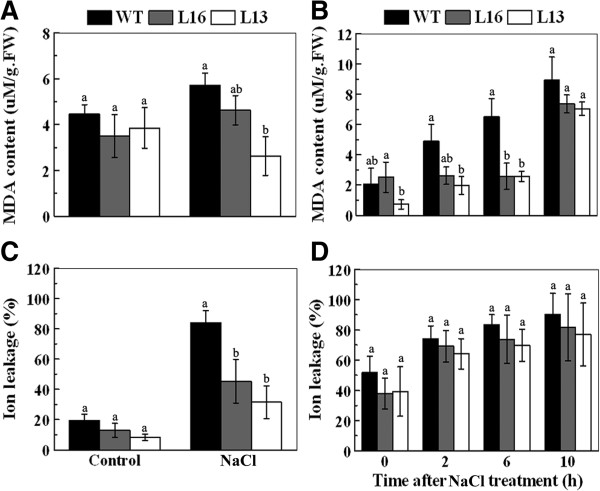
**Physiological analyses of WT and *MaPIP1;1*-overexpressing transgenic lines under salt treatment.** Malonaldehyde content **(A, B)** and ion leakage **(C, D)**, measured on leaf strips **(A, C)** and whole seedlings **(B, D)** of control and transgenic *Arabidopsis* plants under normal conditions and salt treatment. Data are means ± SE of n = 4 biological replicates. Means denoted by the same letter do not significantly differ at *P* < 0.05 as determined by Duncan’s multiple range test.

Increased salt tolerance in transgenic lines relative to the WT led us to investigate physiological differences between *MaPIP1;1* overexpression lines and WT plants. Malonaldehyde (MDA) is a product of lipid peroxidation caused by reactive oxygen species (ROS), and is used to evaluate ROS-mediated injury in plants [31]. The MDA content was lower in transgenic seedlings and in the leaves of the *MaPIP1;1* transgenic lines compared to WT under salt conditions (Figure 5A and 5B). Ion leakage (IL), an important indicator of membrane injury, exhibited a pattern similar to MDA content in leaves and was also lower in the transgenic lines compared to WT under salt conditions (Figure 5C). These results suggest that *MaPIP1;1* overexpression lines experienced less lipid peroxidation and membrane injury under salt stress.

### Overexpression of *MaPIP1; 1* decreases K^+^ and Na^+^ accumulation and increases the K^+^/Na^+^ ratio under salt stress

**Figure 6:**
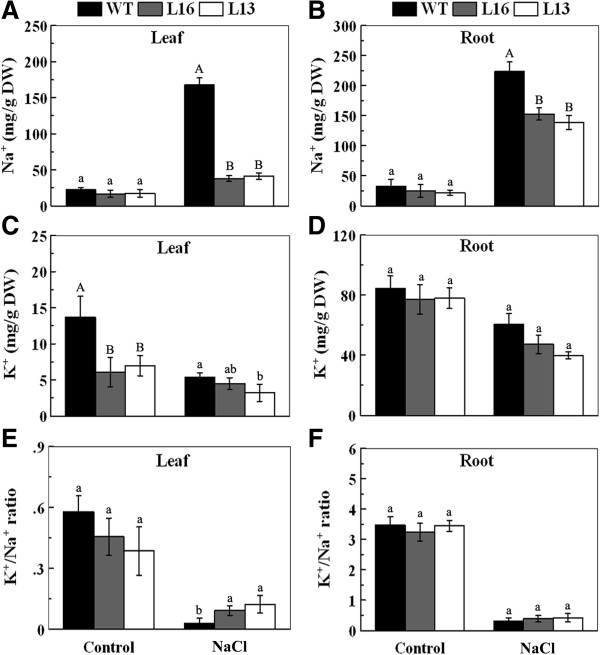
**Ion content in leaves (A,C,E) and roots (B,D,F) sampled from WT and *MaPIP1;1*-overexpressing transgenic lines.** Data are means ± SE of n = 4 biological replicates. Means denoted by the same letter do not significantly differ at *P* < 0.05 as determined by Duncan’s multiple range test.

Under highly saline conditions, plant cells retain a high cytosolic K^+^/Na^+^ ratio in order to survive [32]. To investigate whether *MaPIP1;1* influences the cellular K^+^/Na^+^ ratio, the K^+^ and Na^+^ contents in the roots and leaves of transgenic lines and WT plants were examined in standard conditions and after salinity treatment (Figure 6). The accumulation of K^+^ in leaves was reduced in the transgenic lines compared to WT under normal conditions. However, after salt treatment, K^+^ and Na^+^ were both depleted in the roots and leaves of the transgenic lines in comparison to WT. Moreover, the leaves of the transgenic lines maintained a higher K^+^/Na^+^ ratio than did WT plants during salt treatment. These results suggest that *MaPIP1;1* overexpression decreased accumulation of cellular K^+^ and Na^+^ and improved the K^+^/Na^+^ ratio under salt stress.

### Overexpression of *MaPIP1;1* enhances tolerance to osmotic and drought stresses

**Figure 7:**
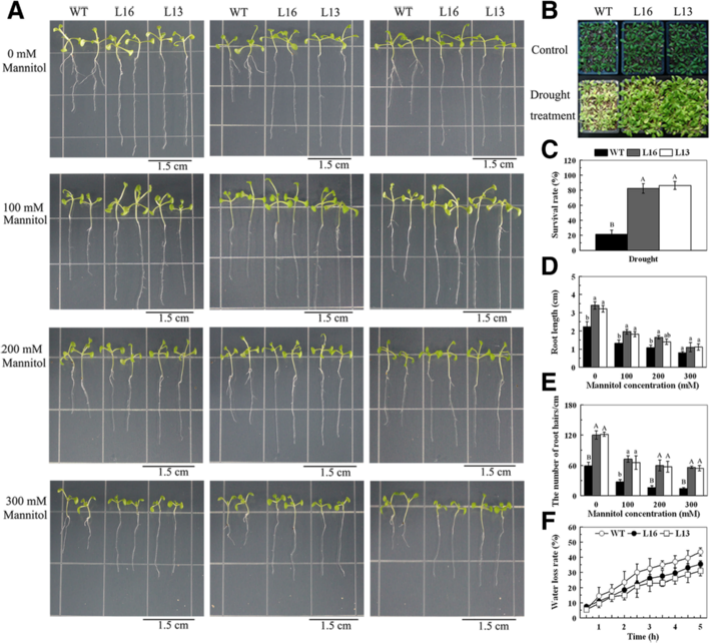
**Response to drought stress of *MaPIP1;1*-overexpressing transgenic *Arabidopsis *plants.** Photographs **(A)** and statistical analyses **(D)** of the primary root length of WT and transgenic lines under normal or osmotic conditions. Photographs **(B)**, survival rates **(C)** and water loss rates **(F)** of WT and transgenic mature plants grown under drought or dehydration conditions. **(E)** The number of root hairs of WT and transgenic lines under normal or osmotic conditions. Data are means ± SE of n = 4 biological replicates. Means denoted by the same letter do not significantly differ at *P* < 0.05 as determined by Duncan’s multiple range test.

To examine the osmotic tolerance of *MaPIP1; 1*-overexpressing transgenic plants, mannitol treatment was applied to transgenic and WT seedlings. The transgenic lines exhibited longer primary roots and more abundant root hairs than WT seedlings with or without mannitol treatment (Figure 7A, 7D and 7E). To determine whether *MaPIP1;1* plays a role in drought stress, transgenic plants and WT *Arabidopsis* plants were subjected to drought treatment. The transgenic plants displayed better growth, more green leaves, higher survival rates and lower water loss rate compared to WT under drought conditions (Figure 7B, 7C and 7F). These results indicate that overexpression of *MaPIP1;1* improved tolerance to drought and osmotic stresses.

### Overexpression of *MaPIP1; 1* reduces IL and MDA content, and increases proline accumulation and osmotic potential under drought stress

**Figure 8:**
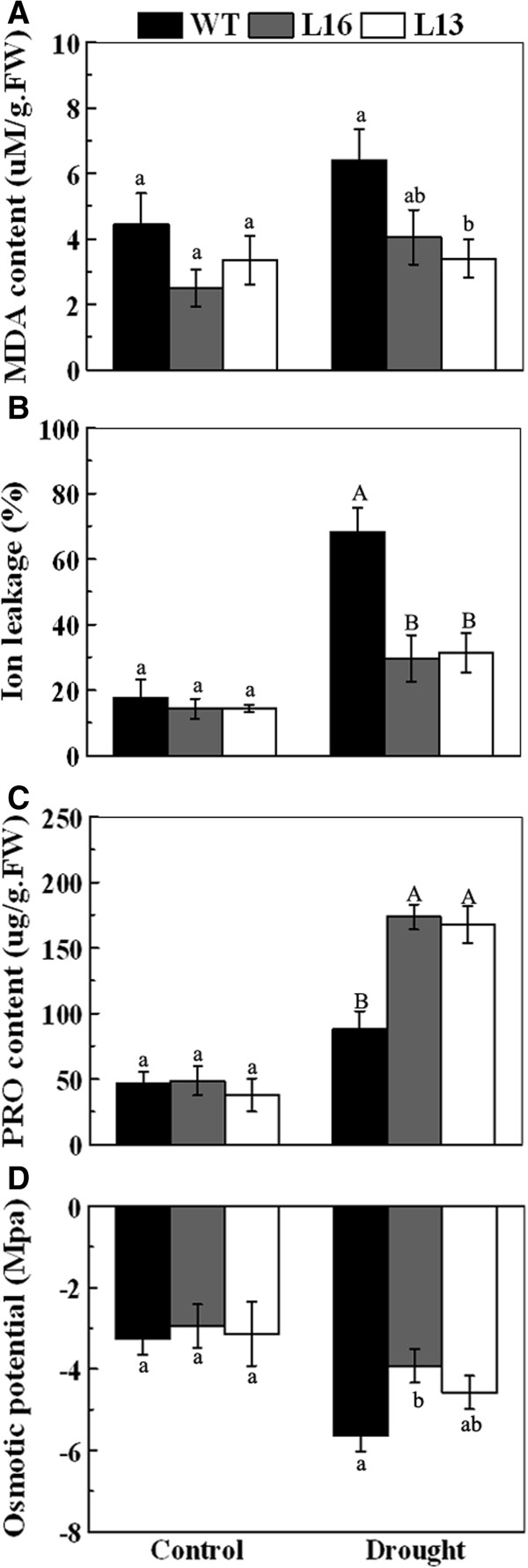
**Physiological analyses of WT and *MaPIP1;1*-overexpressing transgenic lines under drought treatment.** Malonaldehyde content **(A)**, ion leakage **(B)**, proline content **(C)** and osmotic potential **(D)** measured on leaf strips of control and transgenic *Arabidopsis* plants under normal or drought conditions. Data are means ± SE of n = 4 biological replicates. Means denoted by the same letter do not significantly differ at *P* < 0.05 as determined by Duncan’s multiple range test.

Drought stress leads to oxidative injury and disruption of osmotic balance. To investigate the function of *MaPIP1;1* in these physiological processes, IL, MDA, proline and osmotic potential were quantified in the transgenic lines and WT plants under normal and drought conditions. Although no difference in MDA, IL, proline content and osmotic potential was observed in the transgenic lines compared to WT under normal growth conditions, reduced MDA and IL and higher proline content and osmotic potential were observed in leaves of transgenic lines compared to WT under drought treatment (Figure 8A- 8D). These results indicate that the transgenic lines experienced less lipid peroxidation and membrane injury, and improved osmotic adjustment under drought treatment.

### Overexpression of *MaPIP1;1* decreases the expression of ABA-responsive genes

**Figure 9:**
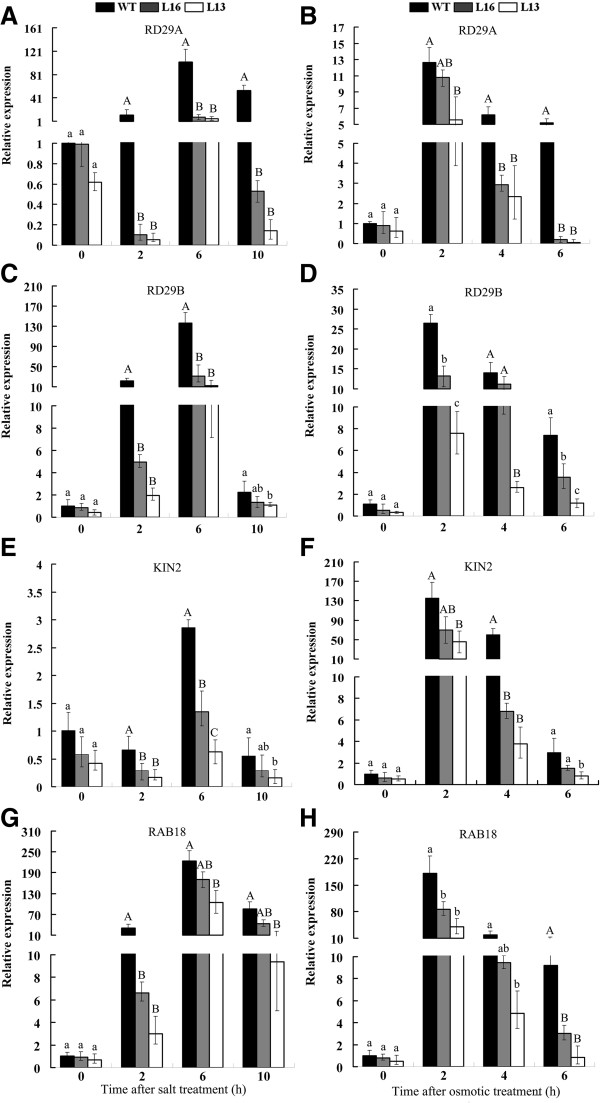
**Expression of ABA-responsive genes in WT and *MaPIP1;1*-overexpressing transgenic lines during salt (A,C,E,G) or osmotic (B,D,F,H) treatments.** Data are means ± SE of n = 3 biological replicates. Means denoted by the same letter do not significantly differ at *P* < 0.05 as determined by Duncan’s multiple range test.

To gain a deeper understanding of *MaPIP1;1* function in abiotic stress tolerance, the expression of several ABA-responsive genes, namely *RD29a*, *RD29b*, *RAB18* and *KIN2* was examined in WT plants and the *MaPIP1;1*-overexpression lines [33-35] (Figure 9). Under standard growth conditions, we observed no significant difference in the transcription of tested genes in the transgenic lines compared to WT plants. However, transgenic seedlings exposed to 2, 4, 6 or 10 h of dehydration or salt treatment exhibited reduced expression of *RD29a*, *RD29b*, *RAB18* and *KIN2* compared to WT seedlings that were similarly treated. These results indicate that *MaPIP1;1* overexpression leads to downregulated expression of ABA-responsive genes during dehydration and salt stresses.

## Discussion

### *MaPIP1;1* plays a positive role in mediating drought and salt stress responses

Several lines of evidence have shown that AQPs are involved in abiotic stress tolerance [11,13,22,25,26,36]. In our study, we observed that expression of *MaPIP1;1* in leaves and roots was significantly induced after drought and salt treatment, implying that this gene product may play a positive role in mediating responses to drought and salt stresses. To better understand the function of *MaPIP1;1* during abiotic stress, we generated a number of *MaPIP1;1-*overexpressing *Arabidopsis* transgenic lines. The transgenic seedlings and adult plants exhibited increased tolerance to drought and salt stresses compared to WT. These results are consistent with previous studies demonstrating that overexpression of *AQP* genes confers abiotic stress tolerance to transgenic plants [11,13,25,26,36].

### Expression of *MaPIP1;1* is associated with reduced membrane injury

Na^+^ is toxic to cell metabolism and has a deleterious effect on some proteins. High Na^+^ levels also reduce photosynthesis and lead to oxidative damage [37]. Additionally, drought stress can induce a rapid accumulation of ROS leading to damage of the cell membrane and oxidation of proteins, lipids, and DNA [38,39]. MDA, the product of lipid peroxidation caused by ROS, can be used to evaluate ROS-mediated injuries in plants [31]. IL is also an important indicator of membrane injury. Thus, MDA content and IL were measured to assess the role of *MaPIP1;1* overexpression in reducing membrane injury under drought or salt conditions. *MaPIP1;1* overexpression resulted in decreased IL and MDA content relative to WT, indicating that *MaPIP1;1*-overexpressing plants may experience less lipid peroxidation and membrane injury under salt or drought conditions. Consistent with our findings, *TaAQP7*-overexpressing tobacco plants show lower levels of MDA and IL when compared to WT under drought stress and *BjPIP1*-overexpressing plants exhibit reduced MDA and IL under Cd stress [26,40]. Overexpression of *OsPIP2;7* in rice results in decreased IL under chilling stress and *TaAQP8*-overexpressing tobacco plants exhibit reduced MDA and IL relative to WT plants under salt stress [25,41]. Collectively these studies indicate that *AQPs* play a vital role in decreasing IL and MDA, thereby reducing membrane injury under various abiotic stresses. AQPs participate in the rapid transmembrane water flow during growth and development in plants. When plants are subjected to drought or salt conditions, increased transport of water across membranes is crucial to maintain a healthy physiological status. We also observed that *MaPIP1;1*-overexpressing plants subjected to drought or salinity treatments exhibited better growth than the WT plants. We surmise that physiological improvements conferred by *MaPIP1;1* overexpression contribute to plants maintaining the protein machinery and hence reducing membrane injury.

### Overexpression of *MaPIP1;1* improves ion distribution under salt conditions

A large number of different ion transporters and channel proteins, such as SOS1, NHX and HKT, are situated in the plasma membrane. These proteins play crucial roles in maintaining ion homeostasis during a variety of abiotic stresses. For example, AtSOS1 and SlSOS1 are membrane-bound Na^+^/H^+^ antiporters that improve salt stress tolerance by exporting Na^+^[42]. The reduced membrane injury observed in *MaPIP1;1*-overexpression lines led us to examine K^+^ and Na^+^ accumulation in WT plants and transgenic lines. *MaPIP1;1* overexpression decreases the accumulation of cellular K^+^ and Na^+^ and improves the K^+^/Na^+^ ratio under salt stress. Previous studies have also reported that aquaporins regulate the distribution of Na^+^ and K^+^ under salt stress. *TaNIP*-overexpressing plants exhibit higher K^+^ and lower Na^+^ levels compared to WT plants under salt stress [13]. *TaAQP8*-overexpressing tobacco plants have elevated Na^+^ and K^+^ levels in roots, reduced Na^+^ and increased K^+^ in stems compared to WT plants under salt treatment [25]. Although overexpression of aquaporins appears to cause different patterns of altered Na^+^ and K^+^ distribution, the evidence suggests that these lead to improved K^+^/Na^+^ ratios under salt conditions. In recent years, a high cytosolic K^+^/Na^+^ ratio has become an accepted marker of salinity tolerance [32]. Therefore, the increased salt stress tolerance conferred by *MaPIP1;1* overexpression may be due to not only decreased membrane injury but also the increased K^+^/Na^+^ ratio in transgenic lines.

### Overexpression of *MaPIP1;1* improves osmotic adjustment under drought conditions

Maintaining the ability to retain water is vital for plants to combat drought stress. AQPs function in rapid transmembrane water flow during growth and development and play important roles in maintaining plant water relations under drought conditions. We observed that *MaPIP1;1*-overexpressing plants exhibited better growth and a lower rate of water loss compared to WT plants under drought conditions, indicating a positive influence of *MaPIP1;1* on water retention. Consequently, we investigated the physiological mechanisms involved in improved water retention conferred by *MaPIP1;1*. When plants experience drought conditions, the accumulation of compatible osmolytes is employed as a strategy to maintain osmotic adjustment. One such compatible solute is the amino acid proline, whose accumulation functions to decrease the cellular osmotic potential and to enhance cellular protection [43]. *MaPIP1;1*-overexpressing transgenic plants maintained higher levels of proline and osmotic potential compared to WT plants subjected to similar drought treatment, implying that *MaPIP1;1* may function in maintaining osmotic adjustment under drought stress. The reduced membrane injury conferred by overexpression of *MaPIP1;1* may also contribute to improved osmotic adjustment under drought stress.

### Reduced expression of ABA-responsive genes in *MaPIP1;1*-overexpressing plants reflects their improved physiological status

Dehydration can lead to inhibition of physiological processes; therefore plants initiate adaptive mechanisms to survive osmotic stresses [44,45]. ABA-dependent signal transduction pathways play crucial roles in the adaptation of plants to stress [33]. When *Arabidopsis* plants were subjected to water stress, some ABA-responsive genes, such as *RD29A*, *RD29B*, *KIN2* and *RAB18* showed increased transcript levels, indicating that the injury resulted from water stress induces the expression of ABA-responsive genes [46]. We examined the expression of these ABA-responsive genes in *MaPIP1;1*-overexpressing transgenic seedlings in relation to WT seedlings. The ABA-responsive genes were downregulated in the transgenic seedlings subjected to dehydration or salt treatments in comparison to similarly treated WT seedlings. This result suggests that the *MaPIP1;1*-overexpressing transgenic plants were less responsive to ABA signaling compared to WT plants, implying that *MaPIP1;1*-overexpressing plants have improved physiological status under drought and salt stress conditions.

## Conclusions

The findings of this study demonstrated a role for *MaPIP1;1* function in improving tolerance to drought and salt stresses. *MaPIP1;1* overexpression resulted in enhanced tolerance to salt stress not only by reducing membrane injury but also by maintaining a higher cellular K^+^/Na^+^ ratio. Enhanced drought stress tolerance conferred by *MaPIP1;1* is related to decreased membrane injury and improved osmotic balance. These findings further our understanding of the mechanisms of environmental stress on plants and highlight the role of *AQPs* in reducing membrane injury, improving ion distribution and maintaining osmotic balance. It is necessary to point out that heterologous expression of banana *MaPIP1;1* in *Arabidopsis* results in these conclusions that are valid for such a heterologous system, but may not be the same in other plants. Further studies are required to characterize the function of *MaPIP1;1* in banana.

## Methods

### Plant materials and growth conditions

Young banana (*Musa acuminata* L. AAA group, cv. Brazilian) seedlings were obtained from the banana tissue culture centre (Danzhou, Institute of Banana and Plantain, Chinese Academy of Tropical Agricultural Sciences). Banana seedlings were grown in soil supplied with half-strength Hoagland’s solution under greenhouse conditions (28°C; 200 μmol m^−2^ s^−1^ light intensity; 16 h light/8 h dark cycle; 70% relative humidity). Seedlings with uniform growth at the five-leaf stage were selected for stress treatment. For NaCl treatment, banana seedlings grown in soil were irrigated with half-strength Hoagland’s solution supplemented with 350 mM NaCl for up to 6 h [47]. Hsiao (1973) proposed that extent of drought stress that plant suffered can be divided to three levels according the water potential in soil [48]. For drought stress assays, water was withheld from banana seedlings grown in soil and samples were collected when the soil moisture content reached different stress degrees as outlined by Hsiao (1973). The soil moisture content was measured using an instrument according to manual instructions (TZS-1, TOP, Zhejiang, China). For low temperature treatments, banana seedlings were transferred into a growth chamber, in which the temperature was maintained at 28, 15, 10, 7 or 5°C for 12 h.

### Cloning of a full-length cDNA encoding banana MaPIP1;1

The full-length cDNA encoding *MaPIP1;1* was amplified by RACE using sequence information from a cDNA fragment previously identified by suppression subtractive hybridization (SSH) [30]. Single-stranded cDNA was used as a source template and was generated from banana fruit 2 d after harvest. For 5′ RACE, the forward primer sequence was 5′-catctcgccgaggtgctccttgtgc-3′ and the reverse primer sequence was 5′-ccttgcctcaacaacacgatc-3′. For 3′ RACE, the forward primer sequence was 5′- cagcggtggcggttggcagcggaggc-3′, and the reverse primer sequence was 5′-ctccgagatctggacgagc-3′. Amplified products were inserted into the pGEM-T easy vector (Promega, Madison, WI, USA). A pair of specific primers was used (5′- tcggccattacggccgggga-3′ and 5′-cttatttttaagggtttttgatac-3′) to amplify the entire open reading frame (ORF) based on the sequences of the 5′ and 3′ ends. The resulting full-length cDNA encoding MaPIP1;1 was assessed by DNAMAN software and BLAST (http://blast.ncbi.nlm.nih.gov/Blast.cgi).

### Subcellular localization of the MaPIP1;1 protein

The MaPIP1;1 ORF, including engineered *Nco*I/*Spe*I restriction sites, was obtained using gene-specific primers. The PCR products were inserted into pCAMBIA1304-GFP expression vector to generate a MaPIP1;1-GFP fusion protein under the control of the CMV35S promoter. The pCAMBIA1304-MaPIP1;1-GFP construct and the pm-rk used as a plasma membrane-localized maker were transiently co-expressed in onion epidermal cells using a gene gun to deliver the expression plasmids (PDS-1000, BIO-RAD) [49]. After a 48 h incubation at 25°C on Murashige and Skoog medium (MS), fluorescence was examined by fluorescence microscopy (LSM700, Carl Zeiss, Germany). The exitation/emission wavelengths are 485/515 nm for GFP, 585/615 nm for RFP and 460/490 nm for GFP and RFP in the same well.

### Plant transformation and generation of transgenic plants

The pCAMBIA1304-MaPIP1;1-GFP construct was transferred into *Agrobacterium* strain GV3101. Transgenic *Arabidopsis* plants were generated using the floral dip-mediated infiltration method [50]. Seeds from T_0_ transgenic plants were selected on half-strength MS medium containing 50 mg/L of hygromycin B. Homozygous T_3_ lines were used for further functional investigation of *MaPIP1;1*. Five hygromycin-resistant transgenic lines from the T_3_ generation were used to determine the integration of *MaPIP1;1* to *Arabidopsis* genome by Southern analysis. The transcriptional levels of *MaPIP1;1* in the 5 independent T_3_ lines was examined by qRT-PCR analysis, in which the *AtActin* gene was used as an internal control.

### Drought and salt stress treatments in WT and transgenic plants

*Arabidopsis thaliana* ecotype Columbia (Col-0) was used as the wild-type control for these experiments. Seeds were sterilized in 75% (v/v) ethanol for 10 minutes, vernalized for 2 d at 4°C in the dark and then germinated on half-strength MS medium or directly on soil. Plants were grown under a chamber (22°C; 120 μmol m^−2^ s^−1^ light intensity; 16 h light/8 h dark cycle; 70% relative humidity). For phenotype analysis in early seedlings under normal conditions, four day-old seedlings were used to determine the root hairs, then the seedlings were transferred to half-strength MS medium for 15 days, and then the photos were taken and roots length were measured. For salt stress tolerance analysis in early seedlings, four day-old seedlings were transferred to half-strength MS or the same medium supplemented with 50–150 mM NaCl for 15 days, then the photos were taken, and then root length and root hairs were measured. For salt stress tolerance analysis in adult plants*, Arabidopsis* plants at four weeks of age were irrigated with 350 mM NaCl for 15 days, then the photos were taken, and then survival rates were assessed. For osmotic stress tolerance analysis, four day-old seedlings were transferred to half-strength MS or the same medium supplemented with 100–300 mM mannitol for 15 days, then the photos were taken, and then root length and root hairs were measured. For drought stress tolerance analysis, plants were grown in pots filled with a mixture of soil and sand (3:1) at 22°C for four weeks. Water was withheld from the treatment group for 20 days, then the photos were taken and survival rates were calculated. For expression analysis of ABA-responsive genes in WT and transgenic lines, fifteen day-old seedlings were transferred to half-strength MS agar plates supplemented with 350 mM NaCl for up to 10 h or 300 mM mannitol for up to 6 h. Whole seedlings were used to quantify relative gene expression.

### Rate of water loss

Thirty fully expanded leaves from each line were detached from four week-old *Arabidopsis* plants and weighed immediately (Fresh Weight, FW). The leaves were placed on open Petri dishes, which were then placed in an incubation chamber (humidity 45%, 22°C). Samples were weighed at different time intervals (Desiccated Weights, DW). The water loss rates was calculated according to the formula: water loss rate (%) = (FW – DW) / FW × 100 [51].

### Ion leakage, proline, malondialdehyde and osmotic potential measurements

Four week-old plants were well watered or irrigated with 350 mM NaCl treatment for 15 days and leaf samples were collected to examine MDA and IL. Fifteen day-old seedlings were transferred to fresh half-strength MS agar plates supplemented with or lacking 350 mM NaCl and whole seedlings were used to measure MDA and IL. Four week-old plants were either well-watered or subjected to simulated drought treatment by withholding water for 20 days. Leaves of WT and transgenic lines were collected to examine MDA, IL, proline and osmotic potential. Ion leakage (IL) was measured according to the method described by Jiang and Zhang (2001) [52]. Leaf samples were cut into strips and incubated in 10 ml of distilled water at 25°C for 8 h. The initial conductivity (C1) was determined with a conductivity meter (DDBJ-350). The samples were then boiled for 10 min to yield complete IL. After cooling down, the electrolyte conductivity (C2) was measured. IL was calculated according to the equation: IL (%) = C1/C2 × 100. Proline content was measured according to Bates (1973) [53]. Malondialdehyde (MDA) content was measured according to the thiobarbituric acid colorimetric method as described by Heath and Packer (1968) [54]. The osmotic potential was measured using a dewpoint PotentiaMeter according to the manufacturer’s instruction (WP4C, DECAGON, USA).

### Measurement of Na^+^ and K^+^ contents

Four week-old *Arabidopsis* plants were well watered or irrigated with 350 mM NaCl for 15 days. The leaves and roots from WT plants and the transgenic lines were collected to determine ion content. Plant materials were washed with ultrapure water, then treated at 105°C for 10 min and baked at 80°C for 48 h. Samples consisting of 50 mg of dry material were dissolved in 6 ml of nitric acid and 2 ml H_2_O_2_ (30%) and then heated at 180°C for 15 min. The digested samples were diluted in a total volume of 50 ml with ultrapure water, transferred into new tubes and analyzed by atomic absorption spectroscopy (Analyst 400, Perkin Elmer, USA).

### Southern blot analyses

Genomic DNA isolated from *Arabidopsis* leaves was digested with *EcoR*I restriction enzyme, separated on 0.8% agarose gels, and transferred to nylon membranes. cDNA probes used in Southern blotting were amplified using a *MaPIP1;1* primer set: F- 5′ATGTGTAATCCCAGCAGC and R- 5′CAAGGAGGACGGAAACAT. The probe was labeled using random primer labeling system. Hybridizations were performed according to the manufacturer’s instructions (Roche11745832910, DIG High Prime DNA Labeling and Detection Starter Kit, USA).

### qRT-PCR

Expression of *MaPIP1;1* in banana organs and leaves after various treatments as well as ABA-responsive genes in *Arabidopsis* were measured by qRT-PCR using SYBR® Premix Ex Taq™ (TaKaRa) chemistry on a Stratagene Mx3000P (Stratagene, CA, USA) instrument. Total RNA was extracted from *Arabidopsis* and banana tissues using a plant RNA extraction kit (QIAGEN) according to the manufacturer’s instructions. 3 μg of total RNA from each sample was converted into cDNA using SuperScript II reverse transcriptase (Invitrogen). In all qRT-PCR experiments, 2^–ΔΔCt^ method was employed to assess relative expression of the tested genes with three replicates of each condition [55]. Prior to quantification experiments, a series of template and primer dilutions were conducted to obtain the optimal template and primer concentrations for amplifying the target genes. Primers used in qRT-PCR analysis had high efficiency and specificity based on melting curve analysis and agarose gel electrophoresis. The sequences of these primers were included in Additional file 1: Table S1. To confirm the specificity of primer pairs, PCR products were subsequently subjected to sequence analysis. Amplification efficiencies of primer pairs were between 90% and 110%. *MaRPS2* (HQ853246) and *MaUBQ2* (HQ853254) were used as the internal controls to normalize expression of target genes in banana and *β-ACTIN2* (At3g18780) and *β-ACTIN8* (At1g49240) were selected as reference genes to normalize transcript levels of target genes in *Arabidopsis*. All the selected reference genes were verified to be constitutive expression and suitable to be used as internal controls [56-58].

## Competing interests

The authors declare that they have no competing interests.

## Authors’ contributions

ZQJ and BYX conceived the study. YX, JHL, JBZ, CHJ and HXM performed the experiments and carried out the analysis. WH designed the experiments and wrote the manuscript. All authors read and approved the final manuscript.

## Supplementary Material

Additional file 1: Figure S1 Comparison of MaPIP1; 1 with other known PIP proteins. Six transmembrane-helix are displayed in the box. The most highly conserved amino acid sequences of MIP are marked with double transverse lines. The ‘NPA’ motif is marked with black dots. The accession numbers of these known proteins in GenBank are as follows: QpPIP1;3 (JQ846272), FePIP1;1 (AY663794), VvPIP1;2 (EF364433), GhPIP1;4 (BK007045) and TuPIP1;5 (KD232839). Amino acid sequences are aligned by ClusterX software. **Figure S2.** Phylogenetic analysis of MaPIP1;1 (boxed) with other known AQPs. The full-length amino acid sequences of AQPs from *Arabidopsis* and rice were used to construct the phylogenetic tree by using ClustalX 1.81 and MEGA 3.1 software. **Figure S3.** Photographs of primary root length of WT and transgenic lines under normal conditions. **Table S1.** Primers used for qRT-PCR analysis. Click here for file
