# Supplementary material for: A banana aquaporin gene, MaPIP1;1, is involved in tolerance to drought and salt stresses
Source: BMC Plant Biol. 2014 Mar 8;14:59. doi: 10.1186/1471-2229-14-59 (PMC4015420; doi:10.1186/1471-2229-14-59)
Supplement: Additional file 1: Figure S1 — Comparison of MaPIP1; 1 with other known PIP proteins. Six transmembrane-helix are displayed in the box. The most highly conserved amino acid sequences of MIP are marked with double transverse lines. The ‘NPA’ motif is marked with black dots. The accession numbers of these known proteins in GenBank are as follows: QpPIP1;3 (JQ846272), FePIP1;1 (AY663794), VvPIP1;2 (EF364433), GhPIP1;4 (BK007045) and TuPIP1;5 (KD232839). Amino acid sequences are aligned by ClusterX software. Figure S2. Phylogenetic analysis of MaPIP1;1 (boxed) with other known AQPs. The full-length amino acid sequences of AQPs from Arabidopsis and rice were used to construct the phylogenetic tree by using ClustalX 1.81 and MEGA 3.1 software. Figure S3. Photographs of primary root length of WT and transgenic lines under normal conditions. Table S1. Primers used for qRT-PCR analysis. [file 1471-2229-14-59-S1.doc]

**
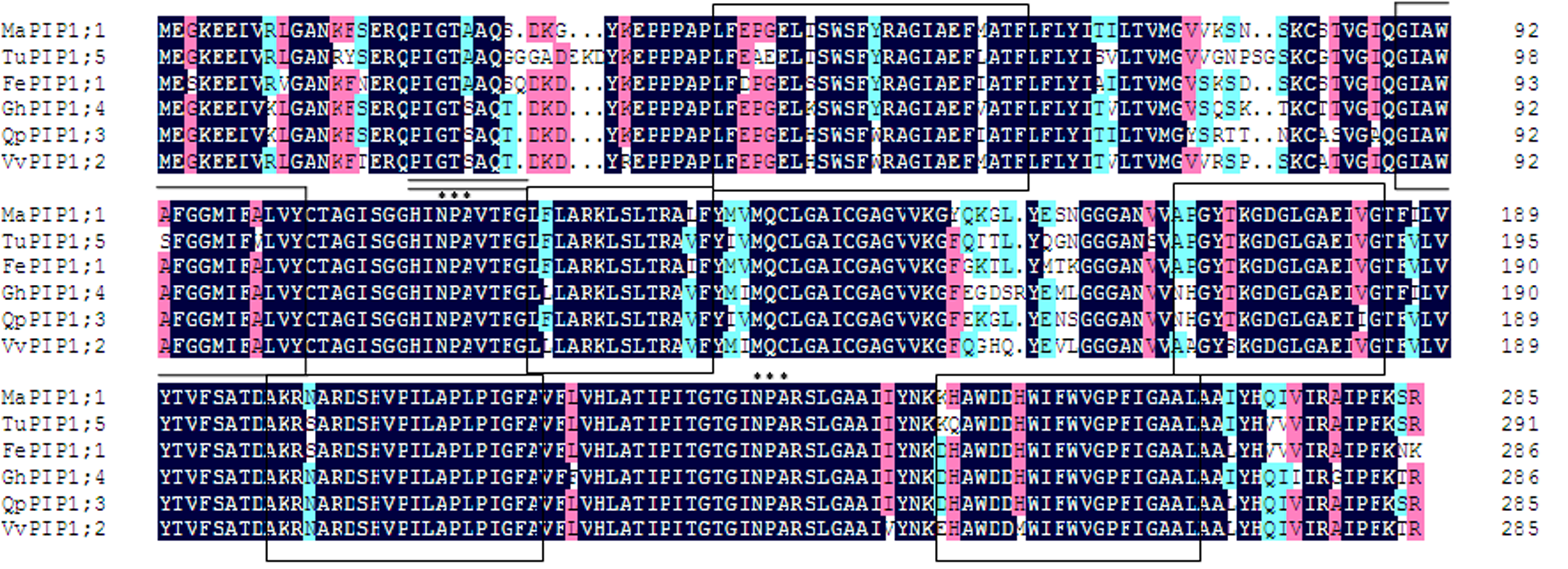
**

**Additional file 1. Comparison of MaPIP1;1 with other known PIP proteins.** Six transmembrane-helix are displayed in the box. The most highly conserved amino acid sequences of MIP are marked with double [transverse lines](http://dict.cnki.net/dict_result.aspx?searchword=横线&tjType=sentence&style=&t=transverse+lines). The ‘NPA’ motif is marked with black dots. The accession numbers of these known proteins in GenBank are as follows: QpPIP1;3 (JQ846272), FePIP1;1 (AY663794), VvPIP1;2 (EF364433), GhPIP1;4 (BK007045) and TuPIP1;5 (KD232839). Amino acid sequences are aligned by ClusterX software.


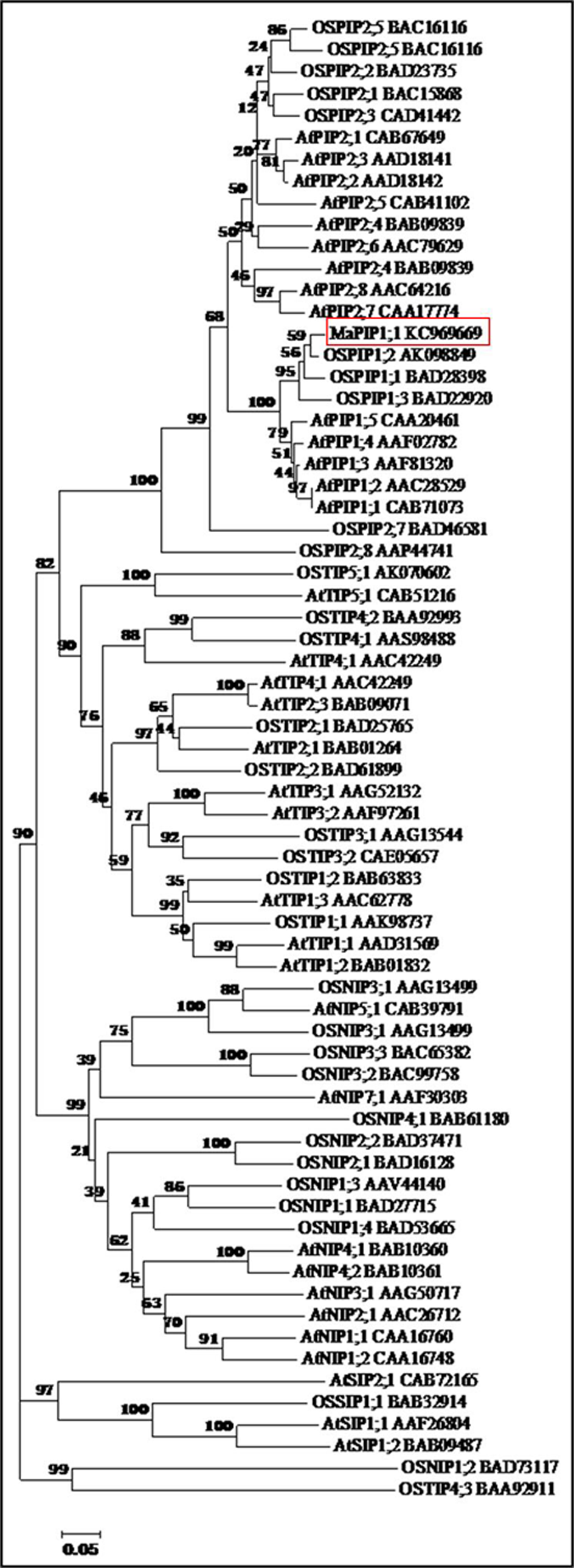


**Additional file 2. Phylogenetic analysis of MaPIP1;1 (boxed) with other known AQPs.** The full-length amino acid sequences of AQPs from Arabidopsis and rice were used to construct the phylogenetic tree by using ClustalX 1.81 and MEGA 3.1 software.


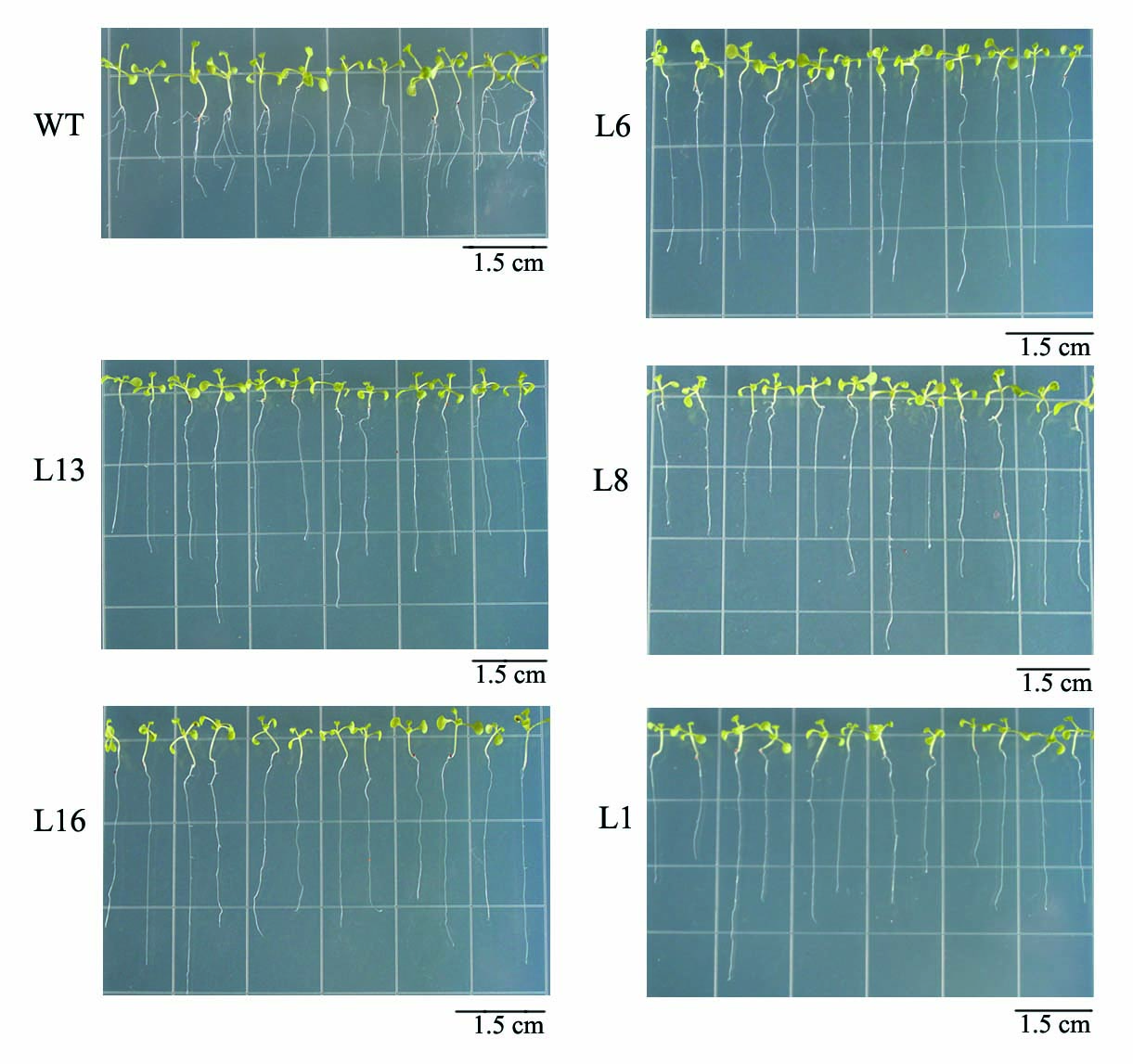
**Additional file 3. Photographs of primary root length of WT and transgenic lines under normal conditions.**

**Additional file 4. Primers used for qRT-PCR analysis.**

| Gene | Forward primer (5’-3’) | Reverse primer (5’-3’) |
| --- | --- | --- |
| *AtRD29A* | GATGGAAGATTCTGTCTCAACGAT | GTTTCTCCTTCACTATCTCCTCCG |
| *AtRD29B* | CGCCACGGTCCGTTGA | TCCACCGGAATCCGAAAAC |
| *AtRAB18* | CCGGTGGTTTACGACAAGAA | CCCAAGCGTTCCAGAGATG |
| *AtKIN2* | GTCAGAGACCAACAAGAATGCC | TGACTCGGATCGCTACTTGTTC |
| *MaPIP1;1* | GCATCACCTTCACCCTCT | CCTTGTCCACCTGGCTAC |
| *β-ACTIN8* | AGTGGTCGTACAACCGGTATTGT | GAGGATAGCATGTGGAAGTGAGAA |
| *β-ACTIN2* | GTACAACCGGTATTGTGCT | ATCAGTAAGGTCACGTCCA |
| *RPS2* | TAGGGATTCCGACGATTTGTTT | TAGCGTCATCATTGGCTGGGA |
| *UBQ2* | GGCACCACAAACAACACAGG | AGACGAGCAAGGCTTCCATT |
